# Psychosocial correlates of eating behavior in children and adolescents: a review

**DOI:** 10.1186/1479-5868-6-54

**Published:** 2009-08-12

**Authors:** Arianna D McClain, Courtney Chappuis, Selena T Nguyen-Rodriguez, Amy L Yaroch, Donna Spruijt-Metz

**Affiliations:** 1 Institute for Health Promotion and Disease Prevention Research, University of Southern California Keck School of Medicine, Alhambra, CA USA; 2 Department of Psychology, University of Southern California, Los Angeles, CA USA; 3 Center for Human Nutrition, Omaha, NE USA

## Abstract

**Background:**

Understanding the correlates of dietary intake is necessary in order to effectively promote healthy dietary behavior among children and adolescents. A literature review was conducted on the correlates of the following categories of dietary intake in children and adolescents: Fruit, Juice and Vegetable Consumption, Fat in Diet, Total Energy Intake, Sugar Snacking, Sweetened Beverage Consumption, Dietary Fiber, Other Healthy Dietary Consumption, and Other Less Healthy Dietary Consumption in children and adolescents.

**Methods:**

Cross-sectional and prospective studies were identified from PubMed, PsycINFO and PsycArticles by using a combination of search terms. Quantitative research examining determinants of dietary intake among children and adolescents aged 3–18 years were included. The selection and review process yielded information on country, study design, population, instrument used for measuring intake, and quality of research study.

**Results:**

Seventy-seven articles were included. Many potential correlates have been studied among children and adolescents. However, for many hypothesized correlates substantial evidence is lacking due to a dearth of research. The correlates best supported by the literature are: perceived modeling, dietary intentions, norms, liking and preferences. Perceived modeling and dietary intentions have the most consistent and positive associations with eating behavior. Norms, liking, and preferences were also consistently and positively related to eating behavior in children and adolescents. Availability, knowledge, outcome expectations, self-efficacy and social support did not show consistent relationships across dietary outcomes.

**Conclusion:**

This review examined the correlates of various dietary intake; Fruit, Juice and Vegetable Consumption, Fat in Diet, Total Energy Intake, Sugar Snacking, Sweetened Beverage Consumption, Dietary Fiber, Other Healthy Dietary Consumption, and Other Less Healthy Dietary Consumption in cross-sectional and prospective studies for children and adolescents. The correlates most consistently supported by evidence were perceived modeling, dietary intentions, norms, liking and preferences. More prospective studies on the psychosocial determinants of eating behavior using broader theoretical perspectives should be examined in future research.

## Background

Diets high in fat and sugar, and low in fruit, vegetables and fiber, have been related to obesity, risk for type 2 diabetes [1,2], cardiovascular disease [3-6], and some cancers [7,8]. Dietary habits tend to form early and track from childhood into adulthood [9,10]. Therefore, the promotion of healthy diet in children and adolescents is a priority to help promote health and well-being, prevent future disease, and reduce the current epidemic of pediatric obesity [11].

To develop effective dietary interventions for children and adolescents, it is necessary to understand the factors that determine eating behavior in these populations. Research has repeatedly shown that theory-based interventions that are guided by relevant behavioral theories are more likely to significantly impact dietary behaviors in youth [11-13]. Theory-based research is fundamental to the understanding of health behaviors by providing a framework by which to examine the relationships among constructs [11,14-16], to assess the impact of the various constructs [11,14,17], and to delineate factors and determinants to be studied [11,15,16,18]. Theory adds coherence and effectiveness to research by identifying facilitating situations and relevant processes and guiding timing and sequencing of events [11,15,16,18]. A good theory indicates methods of intervention and evaluation [11,15,16,18].

The current review represents an updated comprehensive review of psychosocial correlates of a broad range of eating behaviors among children and adolescents. Earlier reviews in youth and adults have focused mainly on determinants of fruit and vegetable intake [19,20], healthy eating [21,22], or environmental factors related to diet [23,24]. For instance, Rasmussen et al [19] recently published a review that examined a broad range of correlates, including psychosocial and environmental correlates, focused exclusively on fruit and vegetable intake. However, recent science has shown that sugar and fiber intake is closely related to insulin dynamics and body composition [25,26]. Sugar sweetened beverage intake is associated with weight gain [27]. Dietary fat has also been shown to have an effect on body fat and on total energy intake [28]. Psychosocial determinants that predict dietary behavior in children and adolescents are not necessarily the same as those that predict dietary behavior in adult populations [11]. Psychosocial as well as dietary intake measures that have been validated in adults are not necessarily valid in younger populations [11]. Some research has suggested that less cognitively-based, more emotionally-based determinants drive adolescent health-related behavior [11,29]. For adolescents and young adults, the immediate satisfaction of psychological needs and adherence to the individual's personal meanings have been shown to be strong motivators and determinants of health behaviors in this age group [30,31]. Research has shown that these age-related differences may be related to neurological development [32].

Therefore, we conducted a systematic review of psychosocial correlates of dietary behaviors in children and adolescents, and included not only fruit and vegetable consumption, but also fat intake, total energy intake, sugar snacking, sweetened beverage consumption, fiber intake, other healthy dietary consumption, and other less healthy dietary consumption. Psychosocial factors are constructs defined here as referring to internal processes in interaction with (but not including) the social environment, and is often used in the context of psychosocial interventions that point towards solutions for individual challenges *within the context *of the social environment. Socio-demographic and environmental correlates were not reviewed. Our review focused on addressing the following two research questions:

a. Which psychosocial correlates of energy, fruit, juice, vegetable, fat, sugar snacking, sweetened beverages, fiber, and other dietary consumption have been studied specifically in youth?

b. Which psychosocial factors are clearly and consistently associated with these dietary behaviors in youth?

## Methods

### Data sources and search strategy

Medical and psychosocial databases (e.g. PubMed, PsychINFO, PsycArticles) as well as references cited in earlier reviews, primary studies and collected articles served to identify potential articles that examined associations between psychosocial factors and dietary intake in pediatric populations. Only papers published in English, between 1990 and May 2009, describing psychosocial factors related to dietary intake were considered for review. No literature searches were conducted after May 27, 2009.

Our search strategy involved using a combination of dietary intake keywords with psychosocial factor keywords to identify relevant articles. For dietary intake, the following keywords were used: eating behavior, dietary behavior, consumption, junk food, high fat food, sodium, fiber, calcium, soda, soft drink, snack food, sugar-laden, beverage, sugar, fast food, sweets, fruit, vegetable, juice, fruit consumption, fruit intake, vegetable consumption, vegetable intake, dietary fat, nutrition, and diet. For psychosocial factors, the following keywords were used: determinants, correlates, self-efficacy, social support, mediation, mediating variables, psychosocial variables, social cognitive theory (SCT), self determination theory (SDT), theory of reasoned action (TRA), theory of planned behavior (TPB), transtheoretical model (TTM), health belief model (HBM), stages of change, SCT, TRA, HBM, SDT, TPB, outcome expectancies, barriers, psychosocial correlates, psychosocial predictors, psychosocial determinants, social theory, psychosocial factors, motivation, knowledge, attitudes, theory, and modeling.

### Inclusion/exclusion criteria

Each study had to meet the following inclusion criteria to be included in this review: people less than 18 years (or mean age within this range) as study sample (special populations were excluded, including pregnant women and athletes); at least one psychosocial variable as an independent variable; a measure of dietary intake (e.g. total energy, fat intake, fruit, vegetable, snack, fast food, calcium, fiber or soft drink consumption) as the dependent variable(s); the papers had to include enough statistical data to be incorporated in the review. Specifically, a correlation coefficient, odds ratio, or beta value as well as a p-value to indicate statistical significance of relationships with a measure of dietary intake were required for inclusion. Because the literature on correlates of eating behaviors in youth is vast, we limited this review paper to cross-sectional and prospective studies. We are currently undertaking a review of determinants of dietary change in intervention studies.

### Identification of relevant studies

Potentially relevant papers were selected by screening the titles (first step), abstracts (second step), and the entire article (third step) retrieved through the database searches. Two researchers (A.M. and C.C.) independently conducted this screening. Disagreement about eligibility between the reviewers was resolved through discussion with a third coauthor (S.R).

### Data extraction

Two authors (A.M. and C.C.) extracted the data from the identified studies. The research design rating presented for each study was developed by using three previously published rating schemes [33-35]. Based on the quality of the research design, each study was given a rating with one to four asterisks. A.M. and C.C. rated the studies and then cross-checked their results. Disagreement between the reviewers was resolved through discussion with S.R. The criteria used to determine the rating of each study is listed in Table 1. Each study's findings and methodological details, such as sample population details, dietary outcomes, psychosocial determinants assessed, assessment methodology (child and/or parent-report, measurement name, reliability, validity), and statistical analysis methods are listed in Tables 2 and 3.

**Table 1:** Study Design Evaluation Criteria

Rating	Description	Criteria
****	Exceptional	-prospective design
		-sample size ≥ 50
		-information on reliability/validity of measures
		-quality of description: population, recruitment, statistical analysis
		-appropriate statistical analyses (regression analysis, controlling for confounders considered exceptional)
***	Strong	-missing 1 of the preceding criteria ^a^
**	Acceptable	-missing 2 of the preceding criteria
*	Weak	-missing 3 or more of the preceding criteria

a: The highest possible rating for cross-sectional/descriptive studies is ***.

**Table 2:** Design and Methodological Characteristics of All Included Articles

Characteristics	Reference Number
**Country**	

Australia	[62,97,43]
Austria	[42,87]*a*
Belgium	[42,87]*c*, [93,96]
China	[90,73]
Denmark	[85,42]
England	[60,95]
Greece	[68,86]
Iceland	[70,42]
Mexico	[79]
Netherlands	[54,74,76,82,96,42,69,110]
Norway	[40,109,42,87]*b*
Portugal	[42]
Spain	[42]
Sweden	[107,41,108,42]
Uganda	[37,102,103]
United States	[38,104,39,44,48,47,106,53,56,61,63,66,67,71,72,75,78,81,83,84,113,88,94,98,111,46,101,55,65,77,59,80,112,92,91]

**Design**	

Experimental	[113,112]
Prospective	[105,104,107,102,103,101,110,111,106,109]
Cross-Sectional	[52,56,60,38,41,48,58,72,85,88,47,51,90,98,57,66,75,84,37,44,50,74,95,100,40,81-83,89,97,71,78,94,46,43,55,65,73,70,69,77,59,80,87,92,91,93,68]

**Sample Size**	

<50	[52,56,113,112]
51–99	[60]
100–499	[38,41,48,58,72,85,88,105,39,47,49,51,106,108,90,98,53,57,61,62,66,75,84,99,111,45,46,102,103,43,55,65,68,59,79,92,91,93]
500–999	[37,104,44,63,64,67,109,74,76,95,100,101,69,80,87]*a*
1000–2999	[40,107,81-83,89,96,97,73,70,86,87]*b*, [87]*c*,[110]
3000–4999	[71,78,50]
>/= 5000	[94,42,77]

**Age Groups**	

Children: (mean age < 13)	[52,56,60,48,47,106,53,82,66,84,111,37,44,95,83,88,96,51,75,61,89,113,64,42,46,55,65,68,73,70,59,79,80,86,112,87,92,91,93,38,58,100]
Adolescents: (mean age > age 13–18)	[99,104,63,109,74,78,39-41,72,105,49,90,98,57,54,62,50,81,97,71,94,85,67,76,107,108,102,103,101,43,69,77,110]

**Ethnicity**	

Asian	[73] (Taiwanese)
Black	[37,51,45,108,102,103]
Hispanic	[38,79]
Native American	[55,65]
White	[49,98]
Diverse	[104,39,44,48,47,50,52,106,53,57,60,63,66,67,71,72,78,81,83,84,88,94,99,111,46,101,77,59,92,91]
Not Reported	[64,61,56,75,80,112,40] (Norwegian), [107] (Sweden), [41] (Sweden), [54] (Netherlands), [62] (Australia), [108] (Sweden), [109] (Norway), [74] (Netherlands), [76] (Netherlands), [82] (Netherlands), [85] (Denmark), [113,95] (British), [96] (Belgium and Netherlands), [97] (Australia), [42] (9 European Countries), [43] (Australia), [68] (Greece), [70] (Iceland), [69] (Netherlands), [86] (Greece), [87] (Austria, Spain, and Norway), [110] (Netherlands), [93] (Belgium)

**Gender**	

Girls Only	[52,66,72,113,45]
Boys Only	[67,59]
Boys and Girls Combined	[37,104,107,44,48,47,50,51,106,53,56-58,60,63,74,78,81-83,85,88,94,111,102,103,42,55,65,68,73,70,69,77,79,112,87,92,91,110-93,62,46,101]
Boys and Girls, Separately	[105,49,84,71,46,101,80,55,100,62,43]

**Instrument for Measuring Food Intake**	

24 hour recalls	[84,50]*a*, [75,100,45,65,70,79]
Food records	[48,47,52,60]*b*, [90,41,53,46,107,83,92,91]
Food Frequency Questionnaire	[60]*a*, [72,49,54,61,66,104,109,74,82,89,96,97,71,78,42,101,43,68,73,69,77,59,86,87,39,40]
Questionnaire (*Unclear whether dietary intake was measured with a FFQ or another questionnaire)*	[38,58,85,105,51,108,98,57,62,99,37,44,50]*b*,[63,64,67,76,95,81,94,102,103,55,80,110,93,88],
Other (*Observation alone or with free-recall and weighed food intake)*	[106,113,56,111,112]

**Validity of Applied Dietary Measure**	

No Information	[56,60,113,38,88,105,57,37,50,76,107,98,85,64,67,71,75,81,82,102,103,43,55,77,79,112,92,110]
Referenced former publications	[48,47,58,61,99,95,54,74,45,46,101,42,65,68,70,69,80,86,87,91,39,63,84,111,44,52,49,51,106,89,83,94,100,41,97,109]
Validity Assessed for Dietary Measure	[104,72,78,40,96,53,66,90,73,59,93]

**Reliability of Applied Dietary Measure**	

No Information	[56,60,38,41,48,85,108,98,57,75,104,50,64,67,74,95,107,82,71,113,102,103,42,73,77,79,112,92,110]
Referenced former publications	[58,101,43,65,68,70,69,80,87,91,39,63,84,44,111,72,52,47,49,51,61,66,94,100,54,62]
< 0.7	**Cronbach's Alpha: **[88,81,86,99] **Interclass Correlation: **[45,83]*fruit*, [83]*veg* **Test-Retest: **[96,78,109]*fat*, [59]*juice*, [59]*veg*, [93]*fruit*, [93]*veg*, [89]*fat*, [89]*fiber*, [89]*fv*
≥ 0.7	**Cronbach's Alpha: **[105,76] **Interclass Correlation: **[46]*fjv* **Test-Retest: **[90,40,97,109]*fv*, [109]*sugar*, [59]*fruit*, **Inter-observer Reliability: **[106,55,37]

**Assessment of Dietary Measure**	

Self-Report	[41,48,58,72,85,88,105,39,47,51,108,90,98,53,57,61,66,75,84,104,44,50,64,74,100,107,81,83,89,96,97,71,78,94,102,103,46,101,43,55,73,70,69,77,59,80,87,92,91,110-93,99,38,52]
Parent-Report	[37,95,82]
Parent- and Self-Report Together	[60,40,65]
Observation and Free Recall	[106]
Parent-Report and Observation	[111]
Weighed Food Intake Observation	[56,113,112]

**Research Design Evaluation Score**	

Exceptional ****	[105,109]
Strong ***	[45,102,42,46,101,55,68,70,59,79,112,87,110,39,82,63,84,99,78,96,44,111,71,104,49,61,66,83,89,106,113,81,75,58,94,100,95,98,41,108,62,97,37,40,74,76]
Acceptable **	[65,73,69,77,80,86,92,91,93,64,72,38,47,51,50,48,53,56,57,67,88,60,54,85,90]
Weak *	[52]

**Table 3:** Evaluation of Dietary Measurement Instrument

	% of time 24 Hour Recall is Used	% of time 24 Hour Recall is Significant	% of time Food Record is Used	% of time Food Record is Significant	% of time FFQ is Used	% of time FFQ is Significant	% of time Other Questionnaire is Used	% of time Other Questionnaire is Significant	% of time Other Measure is Used	% of time Other Measure is Significant
Fruit, Juice and Vegetable Intake	14%	55%	23%	61%	43%	67%	17%	65%	3%	† 100%
Fat Intake	19%	19%	6%	† 50%	44%	28%	19%	75%	13%	100%
Total Energy Intake	20%	30%	7%	† 100%	33%	70%	33%	78%	7%	† 0%
Sugar Snacking	17%	50%	8%	% 43%	25%	44%	42%	57%	8%	† 100%
Sugar-Sweetened Beverages	30%	92%			30%	33%	40%	64%		
Fiber Intake			60%	67%	20%	† 100%	20%	† 100%		
Other Healthy Dietary Intake			21%	69%	50%	55%	29%	45%		
Other Less Healthy Dietary Intake			15%	57%	31%	69%	46%	43%	8%	† 100%

**TOTAL**	**10%**	**50%**	**16%**	**61%**	**35%**	**58%**	**36%**	**57%**	**5%**	**86%**

† 1 article was used to assess this statistic

### Summarizing study findings

Modeling after Sallis' review of the psychosocial determinants of physical activity in children and adolescents [36], associations between psychosocial factors and dietary outcomes were coded as '+' for a positive association and '-' for a negative association. Associations were regarded significant when the p-value reported in the study was <0.05. For studies that reported results from univariate and multivariate analysis, we only reported the multivariate results. The results of multivariate analyses provide a more accurate description of a relationship because they control for other potential confounding variables. Using the above-mentioned criteria, 97 distinct psychosocial constructs were found. In order to reduce the number of variables, we combined conceptually similar psychosocial factors (e.g. appeal of food was combined with preference). Outcome categories were created if there were at least 5 articles that addressed the same specific dietary outcome. This decision rule elicited 8 categories, 6 for specific dietary outcomes and 2 'other' categories: Fruit, Juice and Vegetable Consumption, Fat in Diet, Total Energy Intake, Sugar Snacking, Sweetened Beverage Consumption, Dietary Fiber, Other Healthy Dietary Consumption, and Other Less Healthy Dietary Consumption. Findings are reported below using these categories. Consistent findings were defined as having a relationship in the same direction over 60% of the time as seen in at least two independent articles.

## Results

### Search and selection of studies

The databases search located 4460 titles (Pubmed 3336; PsychInfo 1124) of potentially relevant articles. Other scholarly databases did not yield any new titles. Reference sections of earlier reviews and primary studies added forty-four titles. Screening the titles and abstracts resulted in a selection of 146 articles for full-text review. Sixty-nine of these articles did not meet the inclusion criteria, resulting in a final inclusion of 77 articles.

From the evaluation of the design and methodology of each paper (Table 2) the main findings are as follows:

• Forty-eight (62%) of the included papers were studies conducted in the United States (US).

• Sixty-four (83%) were cross-sectional studies [37-100]. Eleven (14%) were prospective studies [101-111]. Two (3%) were experimental studies [112,113].

• Forty-four (57%) studies had a sample smaller than 500 individuals.

• Forty-three (56%) studies were conducted in children (mean age of less than 13 years); whereas thirty-four (44%) were conducted with adolescents.

• Thirty-five (45%) studies were conducted in ethnically diverse populations. Six (8%) were among African Americans only; two (3%) among Caucasians only; two (3%) among Hispanics only; and thirty (39%) had insufficient information for assessing representativeness.

• To assess dietary behaviors, eight studies used variations of a 24-hour dietary recall; twelve used variations of a food record; twenty-seven used a food frequency questionnaire (FFQ); In a majority of the studies (twenty-seven papers) it was unclear whether dietary intake was measured with a FFQ or another type of questionnaire, such as a diet screener; two studies used observation; three studies used weighed food intake. [Note: This does not equal 77 articles and percentages are not presented because there was overlap (two studies (3%) used more than one measure).]

• Validity and reliability of the dietary intake measure was reported in more than half of the studies. Thirty studies (38%) reported no information on validity. Thirty-seven studies (48%) referenced previous studies. Eleven studies (14%) assessed validity in the article. Twenty-nine studies (38%) reported no information on reliability. Twenty-seven studies (35%) referenced previous studies. Twenty-one studies (27%) assessed reliability in the article.

• Research design evaluation scores were provided for each study. Forty-nine studies (38%) were rated as strong. Twenty-five studies (32%) were rated as acceptable. Only two studies (3%) were rated as exceptional. One study (1%) rated as weak.

• Psychosocial determinants of:

○ fruit, vegetable, and fruit juice intake were examined in 35 studies;

○ fat intake in 16 studies;

○ energy intake in 15 studies;

○ sugar snacking in 12 studies;

○ fiber intake in 10 studies;

○ other dietary consumption (e.g. calcium, healthy dietary behavior, milk intake, fast food) in 14 studies.

○ 13 studies assessed multiple dietary behaviors (e.g., fruit, vegetable, fruit juice and fat) and since there is overlap, percentages are not presented.

Table 3 gives a summary frequency of the use of dietary measures and the percentage of time a dietary measure produced significant findings.

• 35% of studies used a food frequency questionnaire and 36% of studies used other questionnaires (it was unclear whether dietary intake was measured with a food frequency questionnaire or another type of questionnaire such as a food screener).

• Dietary intake as measured by food frequency questionnaire was significantly related to a psychosocial correlate with 58% frequency; dietary intake as measured by other types of questionnaires was related to a psychosocial correlate with 57% frequency. Dietary intake as measured by food records and food recalls were significantly related to a psychosocial correlate with 61% and 50% frequency, respectively.

Tables 4, 5, 6, 7, 8, 9, 10, 11 summarize the associations between potential determinants of dietary intake among children and adolescents. The determinants were grouped by dietary category: fruit, fruit juice, and/or vegetable consumption, fat intake, total energy intake, sugar snacking, sweetened beverage consumption, fiber intake, other "healthy" dietary consumption, and other less healthy dietary consumption.

**Table 4:** Summary of Correlates of Fruit, Juice and Vegetable Intake among Children and Adolescents

Determinant Variable	Association (Reference no.)	No association (Reference no.)
Advanced Stage of Change	Pos assoc: **FV**:[51]	
Attitude	Pos assoc: **F**:[74,42]**V**:[42]	**F**: [70,93]**V**: [70,93]**FV**:[96]
Attitude *(Reported by Parent)*	Pos assoc: **F**:[82]**V**:[82]	
Attitude *of parent*	Pos assoc: **F**:[60]*mother*	**V:**[60]*mother*
Availability	Pos assoc: **F**:[47,93,68,70]*home*, **V**:[47,68,70]*home*,[42]*home*,[59]*home* **FJ**:[59]*home* **FV**:[63,99,78] **FJV**:[46,46]**girls**	**F**: [42]*home*, [2]*school*, [ 42]*friend's home*, [70]*school***V**: [42]*school*, [42]*friend's home*, [70]*school***FV**: [96]*home*,[96]*friend's home*, [96]*school***FJV**:[46]**boys**
Availability *(Perceived by Parent)*	Pos assoc: **F**:[39]	**F: **[45] **V: **[45,39] **FV: **[101]**boys**, [101]**girls**, **FJV:**[46]
Barriers	Pos assoc: **F**:[42] **V**:[42] Neg assoc: **FV**:[96]	**F:**[70] **V:**[70]
Barriers *(Perceived by Parent)*	Neg assoc: **F**:[48] **FJV**:[48]	
Behavioral Skills	Pos assoc: **FV**:[40]	
Family Meal Patterns	Neg assoc: **FV**:[63]	
Family Rules	Pos Assoc: **F**:[70]*demanding* **V**:[70]*demanding*	**F:**[70]*allowing* **V:**[70]*allowing*
Healthful Rules *(Reported by Parent)*	Pos assoc: **FV**:[100,100]**boys**	**FV:**[100]**girls**
Intention *to eat healthy*	Pos assoc: **F**:[87]*a*, [87]*b*, [87]*c* **FV**:[104,96] Neg assoc: **F**:[74,40]	
Intention *(Reported by Parent)*	Pos assoc: **F: **[82] **V: **[82]	
Knowledge	Pos assoc: **F**:[42,70] **V**:[42,70] **FV**:[96,40,97,84]**girls**,	**F**: [60] **V**: [60] **FV**:[83,79,84]**boys**
Knowledge *of parents*	Pos assoc: **F**:[60]*mother*	**V**:[60]*mother*
Liking	Pos assoc **F**:[42,70] **V**:[42,70] **FV**:[96,97]	**F:**[60] **V:**[60]
Modeling	Pos assoc: **F**:[42,70,93]*parents*, [47]*parents*, [93]*peer* **V**:[42,70,93]*parents*, [93]*peer* **FV**:[40,96,97]*parents*, [99]*parents* **FJV**:[47]*parents*	**F**:[74]*mother*, [74]*father*
Modeling *(Reported by Other)*	Pos assoc: **F**:[95,82]*parents*, [82]*mother*, [60]*mother*, [82]*father* **V**:[95,82]*parents*, [82]*mother* **FV**:[95,40]*parents* Neg assoc: **FV**: [52]*parent*	**F**:[101]*parents boys*, [101]*parents girls* **V**:[60]*mother*, [82]*father*, [101]*parents boys*, [101]*parents ***girls**
Motivation	Pos assoc: **FV**:[84]**boys**, [84]**girls**	
Negative Parenting Practices *(Reported by Parent)*	Neg assoc: **F**:[48] **V**:[48] **FJV**:[48]	
Norms	Pos assoc: **FV**:[48,92,63]*family*, [63]*peer* **V**:[92]*total*, [92]*low fat* **FV**:[92] Neg assoc: **FJV**:[47]*peer*	**F**:[74] **V**:[92]*high fat*
Norms *(Reported by Parent)*	Neg assoc: **F**:[82]	**V**:[82]
Outcome Expectations	Pos assoc: **F**:[53]*health* **V**:[53]*social*,[53]*health* **FV**:[53]*social*,[53]*health*, [83]*positive*	**F**: [93,53]*social*, [60]*health* **V**: [93,60]*health* **FV**: [79,83]*negative*
Outcome Expectations *of parents*	Pos assoc: **F:**[60]*mother* Neg assoc:**V:**[60]*mother*	
Parental Control	Pos assoc: **V**:[93]*permissive eating practices*, [93]*obligation rules* Neg assoc: **FV**:[63]*permissive eating practices*	**F**: [93]*permissive eating practices*, [93]*obligation rules* **FV**:[99]
Parental Encouragement	Neg assoc: **F:**[70]*active* **V:**[70]*active*	**FV**:[96]*active*
Parenting Style	Pos assoc: **F:**[69] **FV:**[96]*facilitation*, [96]*demanding*	**FV**:[99]*authoritative*, [96]*allowance*
Perceived Parent Evaluation of Child's Diet as Healthy	Pos assoc: **FV**:[109]	
Preferences	Pos assoc: **F**:[93,70,42,53,93]*in difficult situations* **V**:[93,70,42,53,60,40,59] **FJ**:[59] **FV**:[106,53]*fruit*, [53]*veg*, [63]*veg*, [78,83]*fv*, [53]*fv*	**F**: [79,93]*for healthy eating* **V**: [79,93]*in difficult situations*, [93]*for healthy eating* **FV**:[96]
Preferences *of the child, (Reported by Parent)*	Pos assoc: **F**:[82] **V**:[82] **FV**:[40]	
Regret/Avoid Eating		**FV**:[52]
Self-Competence		**FV**:[52]*social*, [52]*athletic*
Self-Efficacy	Pos assoc: **F**:[92,70,92]*veg* **V**:[82,59,79,70,92]*low fat veg*, [53]*for breakfast and lunch* **FV**:[40,63,88,96,99,53]*for breakfast and lunch* Neg assoc: **V**:[92]*high fat veg*	**F**: [42,74,79,87]*a*, [87]*b*, [87]*c*, [53]*after school*, [53]*breakfast and lunch*, [53]*assisted shopping*, [53]*independent shopping*, **V**: [42,53]*after school*, [53]*assisted shopping *[53]*independent shopping*, **FJ**: [59] **FV**:[53]*after school*, [53]*independent shopping*, [53]*assisted shopping*
Self-Efficacy *(Reported by Parent)*	Pos assoc: **F**:[48,82] **V**:[82]	
Self Evaluation	Pos assoc: **FV**:[109]*of health*, [109]*of diet* Neg assoc:**FV**:[109]*negative*	
Social Desirability	Pos assoc: **FV**:[63]	**F**: [45] **V**: [45,59] **FJ**:[59]
Social Support	Pos assoc: **V**:[42]*parent* **FV**:[96]*parent*, [100]*family total*, [100]*family girls*	**F**: [74,93,42]*parent* **V**: [93] **FV**:[99]*parent*, [100]*family boys*

**LEGEND: F **– Fruit; **J **– Juice; **V **– Vegetables

**Table 5:** Summary of Correlates of Fat Intake among Children and Adolescents

Determinant Variable	Association (Reference no.)	No association (Reference no.)
Attitudes	Neg assoc:[74]	
Availability *(Reported by Parent)*		[45]*fjv*, [39]*veg*, [39]*fruit*, [39]*fat foods*
Conformity to Parents		[49]**boys**, [49]**girls**
Eat to Improve Mood	Pos assoc: [52]	
Healthful Rules *(Reported by Parent)*	Neg assoc: [100]**total**, [100]**boys**	[100]**girls**
Intention	Neg assoc: [104]*to eat healthy*	[74]*to change behavior*
Knowledge		[61]*total*, [61]*saturated*, [75]*total*, [75]*%energy from fat*
Liking Healthy Foods		[75]*total*, [75]*%energy from fat*,
Modeling	Neg assoc: [58]*friend*	[74]*mother*, [74]*father*, [75]*total*, [75]*%energy from fat*
Modeling *(Reported by Other)*	Pos assoc: [54]*mother ***boys**, [54]*mother ***girls**	[52]*parents*, [54]*father ***boys**, [54]*father ***girls**, [54]*friend*
Norms		[74]
Parental Control over Diet	Pos assoc: [111]*general* Neg assoc: [111]*over fat*	
Preferences	Pos assoc: [56]*fat*	[75]*total daily fat*, [75]*%energy from fat*,
Regret/Avoid Eating		[52]
Self-Competence	Pos assoc: [52]*social*	
Self-Efficacy	Neg assoc: [62,49]**boys**, [75]*%energy from fat*,	[74,49]**girls**, [75]*total daily fat*
Social Desirability		[45]
Social Support	Pos assoc: [89]*friend*, [89]*family/friend*	[74,38]*family*, [89]*family*, [100]*family*
Stage of Change	Neg assoc: [58]	

**Table 6:** Summary of Correlates of Total Energy Intake among Children and Adolescents

Determinant Variable	Association (Reference no.)	No association (Reference no.)
Availability	Poy assoc: [44]*home*, [44]*school*	
Availability *(Perceived by Parent)*		[45]*fjv*
Behavioral Skills	Pos assoc: [44]*food preparation*	[44]*food purchasing*
Intention *to eat healthy*	Pos assoc: [105]**boys**, [105]**girls** Neg assoc: [104]	
Knowledge	Pos assoc: [86,44,65,80]*6*^*th*^*grade ***girls**, [80]*6*^*th*^*grade ***boys**, [80]*7*^*th*^/*8*^*th*^*grade ***boys**,	[61,75,80]*7*^*th*^/*8*^*th*^*grade ***girls**,
Liking *healthy foods*	Pos assoc: [75]	
Modeling	Pos assoc: [112]	[75,44]*family*, [44]*media*
Modeling *(Reported by Other)*	Pos assoc: [54]*mother*, [54]*father*, [54]*friends*	
Norms	Pos assoc: [105]*parents ***boys**, [105]*parents ***girls**, [105]*peers ***boys**, [105]*peers ***girls**	
Preferences	Pos assoc: [65]	[44,75,56]*fat*, [45]*sweet beverage*
Self-Efficacy	Pos assoc: [90,86,44]*low fat selection*	[75,49]**girls**, [49]**boys**, [44]*for fv selection*
Social Support	Pos assoc: [86,98]*to eat healthy food* Neg assoc: [44]*to eat fv*	
Social Desirability		[45]*fjv*
Value Expectancy Belief	Pos assoc: [44]	

**Table 7:** Summary of Correlates of Sugar Snacking among Children and Adolescents

Determinant Variable	Association (Reference no.)	No association (Reference no.)
Attitude	Neg assoc: [102,37,85]	
Availability	Pos assoc: [43]**girls**	[43]**boys**
Barriers		[37]
Behavioral Control	Pos assoc: [102]	[103]
Conformity to Parents		[49]**boys**, [49]**girls**
Evaluation of Child's Diet as Healthy *(Reported by Parent)*	Neg assoc: [109]	
Intention	Pos assoc: [103]*to consume sugar snacks and drinks*, [102]*to consume sugar snacks and drinks*, [76]*to consume sugar snacks*	
Lack of Family Conflict	Neg assoc: [43]**girls**	[43]**boys**
Liking	Pos assoc: [60]	
Liking *(As Perceived by Parent)*	Pos assoc: [60]*mother*	
Modeling	Pos assoc: [113]*peer*	
Modeling *(Reported by Parent)*	Pos assoc: [43]*mother ***boys**	[43]*mother ***girls**, [60]*mother*
Norms		[37,45]*fjv*
Knowledge	Neg assoc: [50]	[60,85]
Knowledge *of parent*		[60]*mother*
Outcome Expectations	Neg assoc: [60]*health*	[85]
Outcome Expectations *of parents*		[60]*mother*
Parental Pressure to Eat More Food	Pos assoc: [43]**boys**	[43]**girls**
Parenting Style *(Reported by Parent)*		[43]*authoritarian ***boys**, [43]*authoritarian ***girls**
Perceived Risk	Pos assoc: [37]	
Self-Efficacy *in making healthy food choices*	Neg assoc: [49]**boys**, [49]**girls**	[85]
Self-Evaluation	Neg assoc: [109]*of diet*	[109]*negative*, [109]*of health*

**Table 8:** Summary of Correlates of Sweetened Beverage Intake among Children and Adolescents

Determinant Variable	Association (Reference no.)	No association (Reference no.)
Attitude	Pos assoc: [110]	
Availability	Pos assoc: [64]*home*, [64]*school*	[72,43]**boys**,[43]**girls**
Availability *(Perceived by Parent)*		[45]*of fjv*
Intention	Pos assoc: [67]*to drink soda*, [110]*to drink soda*	
Knowledge	Pos assoc: [97]	
Lack of Family Conflict		[43]**boys**, [43]**girls**
Liking	Pos assoc: [64,97] Neg assoc: [64]*water*	[64]*milk*, [72]*orange juice*
Modeling	Pos assoc: [97]*parents*, [47]*parents*, [64]*parents*, [110]*parents*, [64]*friends*, [110]*friends*, [97]*peers*	
Modeling *(Reported by Other)*	Pos assoc: [43]*mother ***girls**, [43]*mother ***boys**	[43]*father ***girls**, [43]*father ***boys**
Norms	Pos assoc: [64]*peer*, [110]*parent*, Neg assoc: [91]*milk norms*	
Number of Meals Family Eats Together		[38]
Parental Control	Pos assoc: [47]	
Parenting Style *(Reported by Parent)*	Pos assoc: [43]*authoritarian ***boys**	[43]*authoritarian ***girls**
Perceived Behavioral Control		[110]
Preferences		[45]
Self-Efficacy	Pos assoc: [67] Neg assoc: [91]*to drink milk*	
Social Desirability		[45]
Social Support *for healthy eating*		[38]*family*

**Table 9:** Summary of Correlates of Fiber Intake among Children and Adolescents

Determinant Variable	Association (Reference no.)	No association (Reference no.)
Number of Meals Family Eats Together	Pos assoc: [38]	
Intention	Pos assoc: [107]*to eat healthy*	
Knowledge	Pos assoc: [41]*general*, [41]*specific*	
Modeling *(Reported by Other)*		[52]*parent*
Perceived Behavioral Control		[107]
Self-Competence	Pos assoc: [52]*academic*	
Social Support *for healthy eating*	Pos assoc: [38]*family*, [89]*family*, [89]*family/friend*, [89]*friend*	

**Table 10:** Summary of Correlates of "Healthy" Dietary Consumption among Children and Adolescents

Determinant Variable	Association (Reference no.)	No association (Reference no.)
Attitude *towards health*	Pos assoc: C:[41], A:[71]**girls**, B:[81]*8*^*th *^*grade*, B:[81]*11*^*th*^*grade*, B:[55]**girls**, B:[55]**total**, D:[73]*1*^*st *^– *3*^*rd*^*grade*, D:[73]*4*^*th *^– *6*^*th*^*grade*	C:[108], G:[108], N:[108], L:[108], A:[71]**boys**, B:[55]**boys**
Availability	Pos assoc: A:[71]**boys**, A:[71]**girls**, O:[72], Q:[72]	C:[72], R:[72], BB:[72], N:[72]
Barriers	Pos assoc: B:[55]**total**, B:[55]**girls**	B:[55]**boys**
Body Satisfaction		A:[71]**boys**, A:[71]**girls**,
Caring about Nutrition Behavior	Pos assoc: D:[73]*1*^*st *^– *3*^*rd *^*grade*, D:[73]*4*^*th *^– *6*^*th *^*grade*,	
Conformity to Parents	Pos assoc: B:[49]**boys**	B: [49]**girls**
Eating Concerns	Pos assoc: B:[81]*8*^*th *^*grade*	B:[81]*11*^*th *^*grade*
Intention	Pos assoc: C:[108]*to consume high fat milk*, G:[108]*to consume high fat milk* H:[107]*to eat breakfast* I:[107]*to eat breakfast* N:[108]*to consume high fat milk*	L: [108]*to consume high fat milk*, B: [55]*to eat healthy*, B: [55]*to eat healthy ***girls**, B: [55]*to eat healthy ***boys**
Knowledge	Pos assoc: B:[81]*8*^*th*^*grade*, B:[81]***11***^*th*^*grade*, K: [97], C:[97], D:[73]*1*^*st *^– *3*^*rd*^*grade*, D:[73]*4*^*th *^– *6*^*th*^*grade*	A:[66], A:[72], N:[97], L:[97], I:[97], O:[97], C:[41]*general knowledge*, C:[41]*specific knowledge*
Liking	Pos assoc: O:[72], N:[97], L:[97], K:[97], I:[97], O:[97], C:[97]	C:[72], Q:[72], N:[72], R:[72], BB:[72]
Modeling	Pos assoc: A:[72]*father*, K:[97]*parents*, L:[97]*parents*, N:[97]*parents*, I:[97]*parents*, L:[97]*parents*, N:[97]*parents*, O:[97]*parents*, C:[97]*peers*, C:[97]*parents* Neg assoc: A:[72]*siblings*	A:[72]*mother*, A:[72]*friends*, K:[97]*peers*, I:[97]*peers*, O:[97]*peers*
Modeling *(Reported by Other)*	Pos assoc: W: [101]*parents ***girls**	W: [101]*parents ***boys**
Norms	Pos assoc: B:[55]**total**, B:[55]**girls**, B:[55]**boys** C:[91]*milk norms*, I:[91]*milk norms*	C:[108], G:[108], N:[108], L:[108], K:[91]*milk norms*
Parental Presence at Meals		A:[71]**boys**, A:[71]**girls**
Perceived Behavioral Control	Pos assoc: I:[107]	C:[108], G:[108], H:[107], L:[108], N:[108]
Perceived Difficulty	Neg assoc: C:[108], G:[108]	L:[108], N:[108]
Preference	Pos assoc: A:[71]**boys**, A:[71]**girls**	
Self-Efficacy	Pos assoc: A:[66], P:[57], C:[91], I:[91], K:[91], A:[71]**girls**	A:[71]**boys**, B:[49]**boys**, B:[49]**girls**
Social Support	Pos assoc: A:[71]**boys**, A:[66]*family*, A:[72]*father*, A:[72]*mother*, A:[72]*friend*	A:[71]**girls**, A:[72]*sibling*, A:[66]*friend*
Time Available to Eat Breakfast		A:[71]**boys**, A:[71]**girls**

**LEGEND: A **– Calcium; **B **– Healthy Dietary Behavior/Food Pyramid Score; **C **– Milk Intake; **D **– Dietary Quality Score; **E **– Sodium; **F **– Fruit; **G **– Yogurt Intake; **H **– Skimmed Milk: Fat; **I **– Low Fat Milk: Fat; **J **– Medium Fat Milk: Fat; **K **– Full Fat Milk: Fat; **L **– Cereal Intake; **M **– Margarine/Spread Intake; **N **– Bread and/or Toast Intake; **O **– Cheese; **P **– Low Fat Vending Snacks; **Q **– Yogurt; **R **– Green Vegetables; **S **– Inadequate Vegetable Consumption; **T **– Inadequate Fruit Consumption; **U **– Inadequate Dairy Consumption; **V **– Vegetables; **W **– Dairy; **X **– Snacks; **Y **– Butter; **Z **– Ice Cream; **AA **– Fat Snacks; **BB **– Tofu

**Table 11:** Summary of Correlates of Less Healthy Dietary Consumption among Children and Adolescents

Determinant Variable	Association (Reference no.)	No association (Reference no.)
Attitude *towards health*	Pos assoc: X:[110]	M:[108], M:[41]
Availability	Pos assoc: X:[43]**boys**, X:[43]**girls**	Z:[72]
Family Connectedness	Pos assoc: S:[77]*very low*, S:[77]*low*; T:[77]*very low*, T:[77]*low*, T:[77]*moderate*	S:[77]*moderate*
Number of Meals Family Eats Together	Pos assoc: S:[94]*6–7 per week*, S:[94]*4–5 per week*, T:[94]*6–7 per week*, T:[94]*4–5 per week*, U:[94]*6–7 per week*, U:[94]*4–5 per week*,	F:[38]
Intention	Pos assoc:AA:[76]*to consume sugar snacks or drinks*, J:[107]*to eat breakfast*, K:[107]*to eat breakfast*, X:[110]*to consume sugar snacks or drinks*	M:[108]*to consume high fat milk*,
Knowledge	Pos assoc: E:[111], F:[97]	Y:[97], M:[97], M:[41]*general knowledge*, M:[41]*specific knowledge*
Lack of Family Conflict		X:[43]**boys**, X:[43]**girls**
Liking	Pos assoc: M:[97], F:[97], Y:[97]	Z:[72]
Modeling	Pos assoc: X:[110]*parents *X:[110]*friends*, Y:[97]*parents*, Y:[97]*peers*, M: [97]*parents*, AA:[97]*parents*, AA:[97]*peers*	M:[97]*peers*
Modeling *(Reported by Other)*	Pos assoc: X:[43]*mother ***boys**	X:[43]*mother ***girls**
Norms		M:[108], X:[110]*parents*
Parental Influence *in food decision-making*		S:[94], T:[94], U:[94]
Parental Presence		S:[94]*when child leaves for school*, S:[94]*when kid returns home from school*, T:[94]*when child leaves for school*, T:[94]*when kid returns home from school*, U:[94]*when child leaves for school*, U:[94]*when kid returns home from school*
Parenting Style *(Reported by Parent)*		X:[43]*indulgent ***boys**, X:[43]*indulgent ***girls**
Perceived Behavioral Control	Pos assoc: J:[107], K:[107], B:[55]**boys**	B:[55], M:[108], B:[55]**girls**
Perceived Difficulty		M:[108]
Poor School Achievement	Pos assoc: S:[77], T:[77]	
Weight Dissatisfaction	Pos assoc: S:[77], T:[77]	
Weight Perception	Pos assoc: S:[94]*as overweight*, T:[94]*as overweight*, U:[94]*as overweight*	S:[94]*as underweight*, T:[94]*as underweight*, U:[94]*as underweight*
Self-Efficacy	Neg assoc: B:[55], B:[55]**girls**	B:[55]**boys**
Social Support *for healthy eating*	Neg assoc: AA:[38]	
Social Support *(Reported by Parent)*	Pos assoc: X:[43]**girls**	X:[43]**boys**

**LEGEND: A **– Calcium; **B **– Healthy Dietary Behavior/Food Pyramid Score; **C **– Milk Intake; **D **– Dietary Quality Score; **E **– Sodium; **F **– Fruit; **G **– Yogurt Intake; **H **– Skimmed Milk: Fat; **I **– Low Fat Milk: Fat; **J **– Medium Fat Milk: Fat; **K **– Full Fat Milk: Fat; **L **– Cereal Intake; **M **– Margarine/Spread Intake; **N **– Bread and/or Toast Intake; **O **– Cheese; **P **– Low Fat Vending Snacks; **Q **– Yogurt; **R **– Green Vegetables; **S **– Inadequate Vegetable Consumption; **T **– Inadequate Fruit Consumption; **U **– Inadequate Dairy Consumption; **V **– Vegetables; **W **– Dairy; **X **– Snacks; **Y **– Butter; **Z **– Ice Cream; **AA **– Fat Snacks; **BB **– Tofu

### Psychosocial Correlates of Fruit, Fruit Juice, and/or Vegetable Consumption

Thirty-five articles tested for psychosocial correlates of fruit, juice, and vegetable consumption defined as consumption of fruit, fruit juice, and/or vegetables (FJV). In several articles, FJV consumption was broken down into more than one outcome variable. Therefore, we report on variables within studies here. *Intention to eat healthy *was positively associated with FJV consumption in 3 of 5 studies [87,96,104] and for 5 out of 7 variables. *Knowledge *was positively associated with FJV consumption in 6 of 9 articles [40,42,70,84,96,97] and for 8 out of 13 variables. Interestingly in one study [84]*knowledge *was positively associated among girls, but not associated among boys. *Liking *was positively associated with FJV consumption in 4 of 5 articles [42,70,96,97] and for 6 out of 8 variables. *Norms *were positively associated with FJV consumption in 3 of 5 articles [48,63,92], 6 out of 10 variables. *Perceived Modeling *was positively associated with FJV consumption in 8 of 9 articles [40,42,47,70,93,96,97,99] and for 14 out of 16 variables. However, *modeling as reported by parent *did not show any consistent associations. *Preferences *were positively associated with FJV consumption in 11 of 13 articles [40,42,53,59,60,63,70,78,83,93,106] and for 20 out of 26 variables. None of the other psychosocial variables examined, including *attitude*, *availability, perceived barriers, outcome expectations, self-efficacy, social desirability*, and *social support *showed consistent associations with FJV intake. Table 4 summarizes the psychosocial correlates of fruit, juice, and/or vegetable consumption among children and adolescents.

### Psychosocial Correlates of Fat Intake

Sixteen articles examined psychosocial correlates of fat intake (*combined total daily fat, % energy from fat, saturated fat*). Overall, none of the factors examined showed consistent associations with dietary fat intake. *Knowledge *was not significantly associated with dietary fat in two studies [61,75] and 4 out of 4 variables. *Perceived modeling *was not significantly associated with dietary fat in 2 of 3 studies [74,75] and for 4 out of 5 variables. None of the other factors examined showed consistent associations with dietary fat intake, including social support. However, we were unable to assess the significance of many factors due to the low number of studies that investigated psychosocial variables and their effect on fat intake. Table 5 summarizes the psychosocial correlates of fat intake among children and adolescents.

### Psychosocial Correlates of Total Energy Intake

Fifteen articles investigated the psychosocial correlates of total energy intake. Only two variables were consistently significantly positively associated with total energy intake. *Knowledge *was positively associated with total energy intake in 4 of 6 articles [44,65,80,86] and for 6 out of 9 variables. *Social support *was positively associated with total energy intake in 2 of 3 articles [86,98] and for 2 out of 3 variables. None of the other psychosocial variables examined, including *intention to eat healthy, perceived modeling, preferences *or *self-efficacy *showed consistent associations with total energy intake. Table 6 summarizes the psychosocial correlates of total energy intake among children and adolescents.

### Psychosocial Correlates of Sugar Snacking

Twelve articles examined the psychosocial correlates of sugar intake, which is defined as consumption of snacks and high sugar-sweetened foods, such as candy. *Attitude *and *intentions *were the only two variables consistently significantly associated with sugar snacking. In three studies [37,85,102], 3 out of 3 variables, *attitude towards healthy eating behavior *was negatively associated with sugar snacking. *Intention *to consume sugar was positively associated with sugar snacking in all three of the articles in which it was measured [76,102,103] for 3 out of 3 variables. No other factors examined showed consistent associations with sugar snacking due to the small number of articles investigating psychosocial associations with sugar snacking. Table 7 summarizes the psychosocial correlates of sugar snacking among children and adolescents.

### Psychosocial Correlates of Sweetened Beverage Consumption

Ten articles examined the psychosocial correlates of sweetened beverage consumption, which is defined as consumption of sodas, sweetened beverages, and/or juice (alone, not in combination with fruits or vegetables). *Intention *was found to be positively associated with sweetened beverage consumption in two studies [67,110] and for the two variables measured. *Perceived Modeling *was shown to be positively associated with sweetened beverage consumption in four studies [47,64,97,110] and for all seven variables measured. However, *modeling as reported by parent *did not show any consistent associations. *Norms *were associated with sweetened beverages in all the studies, using all variables. *Peer norms *and *parent norms *were positively associated with sweetened beverage consumption in 2 of 3 studies [64,110]. Milk norms were negatively associated with sweetened beverage consumption in 1 of the 3 studies [91]. None of the other variables examined illustrated consistent associations with sweetened beverage consumption. Table 8 summarizes the psychosocial correlates of sweetened beverage consumption among children and adolescents.

### Psychosocial Correlates of Fiber Intake

Five articles evaluated the psychosocial correlates of fiber intake, defined as fiber intake and consumption of high fiber cereal or bread. Both familial and friend *social support *were positively associated with fiber consumption in 2 studies [38,89] and for 3 out of 4 variables. No other factors examined showed consistent associations with fiber intake. Table 9 summarizes the psychosocial correlates of fiber intake among children and adolescents.

### Psychosocial Correlates of Other "Healthy" Dietary Consumption

Fourteen articles examined the psychosocial correlates of healthy types of dietary consumption, including calcium intake, healthy dietary behavior, green vegetable intake, milk intake, yogurt intake, skim milk (<.1% fat), low fat milk (.5% fat), cereal intake (cornflakes, muesli, etc.), bread intake, and consumption of low fat vending snacks. *Norms *were positively associated with healthy dietary consumption in 2 of 3 articles [55,91], 5 out of 8 variables. *Perceived modeling *was positively associated with healthy dietary consumption in two articles [72,97], and for 11 out of 16 variables. *Modeling as reported by parent *did not show any consistent associations. *Social support *was positively associated with healthy types of dietary consumption in 3 out of 3 articles [66,71,72] and for 5 out of 8 variables. This association held true among boys, but not girls [71]. *Self-efficacy *was significantly positively associated with healthy dietary consumption in 4 out of 5 articles [57,66,71,91], 6 out of 9 variables. None of the other variables examined, including *attitude *or *knowledge*, illustrated consistent associations with other healthy dietary consumption. Table 10 summarizes the psychosocial correlates of healthy dietary consumption among children and adolescents.

### Psychosocial Correlates of Other Less Healthy Dietary Consumption

Thirteen articles examined the psychosocial correlates of less healthy types of dietary consumption, including fast food, sodium, medium fat milk (1.5% fat), full fat milk (3% fat), margarine/spread intake, inadequate fruit consumption, inadequate vegetable consumption, and inadequate dairy consumption. *Intentions *to eat a particular less healthy food were positively associated with less healthy dietary consumption in 3 out of 4 studies [76,107,110] and for 4 out of 5 variables. *Perceived Modeling *was positively associated with less healthy dietary consumption for 2 of 2 articles [97,110] and for 7 out of the 8 variables measured. *Modeling as reported by parent *did not show any consistent associations. None of the other variables examined, including *attitude towards health *or *norms*, were consistently associated with less healthy dietary consumption. Table 11 summarizes the psychosocial correlates of unhealthy dietary consumption among children and adolescents.

Table 12 gives a summary review of the most frequently studied psychosocial correlates by dietary outcome.

**Table 12:** Summary of Correlates of Dietary Behavior among Children and Adolescents

	FJV	Fat	Total Energy	Sugar Snacking	SSB	Fiber	Other Healthy Dietary Intake	Other Less Healthy Dietary Intake	Total Summary
**Attitude**	5 articles 8 variables Pos assoc: 3 **38%**	1 article 1 variable Neg assoc: 1 † **100%**		3 articles 3 variables Neg assoc: 3 **100%**	1 article 1 variable Pos assoc: 1 † **100%**		6 articles 14 variables Pos assoc: 8 **57%**	3 articles 3 variables Pos assoc: 1 **33%**	19 articles 30 variables Pos assoc: 17 **57%**
**Availability**	12 articles 26 variables Pos assoc: 15 **‡ 57%**		1 article 2 variables Pos assoc: 2 † **100%**	1 article 2 variables Pos assoc: 1 **50%**	3 articles 5 variables Pos assoc: 2 **40%**		2 articles 8 variables Pos assoc: 4 **50%**	2 articles 3 variables Pos assoc: 2 † **67%**	21 articles 46 variables Pos assoc: 26 **57%**
**Barriers**	3 articles 5 variables Pos assoc: 2 **40%**			1 article 1 variable Pos assoc: 0 **0%**			1 article 3 variables Pos assoc: 2 † **67%**		7 articles 11 variables Pos assoc: 6 **55%**
**Intention**	5 articles 7 variables Pos assoc: 5 **71%**	2 articles 2 variables Neg assoc: 1 **50%**	2 articles 3 variables Pos assoc: 2 † **67%**	3 articles 3 variables Pos assoc: 3 **100%**	2 articles 2 variables Pos assoc: 2 **100%**	1 article 1 variables Pos assoc: 1 † **100%**	3 articles 9 variables Pos assoc: 5 **56%**	4 articles 5 variables Pos assoc: 4 **80%**	20 articles 30 variables Pos assoc: 21 **70%**
**Knowledge**	9 articles 13 variables Pos assoc: 8 **62%**	2 articles 4 variables Pos assoc: 0 **0%**	6 articles 9 variables Pos assoc: 6 **67%**	3 articles 3 variables Pos assoc: 1 **33%**	1 article 1 variable Pos assoc: 1 † **100%**	1 article 2 variables Pos assoc: 2 † **100%**	6 articles 14 variables Pos assoc: 6 **43%**	3 articles 6 variables Pos assoc: 2 **33%**	31 articles 52 variables Pos assoc: 26 **50%**
**Liking**	5 articles 8 variables Pos assoc: 6 **75%**	1 articles 2 variables Pos assoc: 0 **0%**	1 article 1 variable Pos assoc: 1 † **100%**	1 article 1 variable Pos assoc: 1 † **100%**	3 articles 5 variables Pos assoc: 2 **40%**		2 articles 12 variables Pos assoc: 7 **58%**	2 articles 4 variables Pos assoc: 3 † **75%**	15 articles 33 variables Pos assoc: 20 **61%**
**Perceived Modeling**	9 articles 16 variables Pos assoc: 14 **88%**	3 articles 5 variables Neg assoc: 1 **20%**	3 articles 4 variables Pos assoc: 1 **25%**	1 article 1 variable Pos assoc: 1 † **100%**	4 articles 7 variables Pos assoc: 7 **100%**		2 articles 16 variables Pos assoc: 10 **63%**	2 articles 8 variables Pos assoc: 7 **88%**	24 articles 57 variables Pos assoc: 41 **72%**
**Modeling** *(Reported by Other)*	6 articles 17 variables Pos assoc: 10 **59%**	2 articles 6 variables Pos assoc: 2 **33%**	1 article 2 variables Pos assoc: 2 † **100%**	2 articles 3 variables Pos assoc: 1 **33%**	1 article 4 variables Pos assoc: 2 **50%**	1 article 1 variable Pos assoc: 0 **0%**	1 article 2 variables Pos assoc: 1 **50%**	1 article 2 variables Pos assoc: 1 **50%**	15 articles 37 variables Pos assoc: 19 **51%**
**Norms**	5 articles 10 variables Pos assoc: 6 **60%**	1 article 1 variable Pos assoc: 0 **0%**	1 article 4 variables Pos assoc: 4 † **100%**	2 articles 2 variables Pos assoc: 0 **0%**	3 articles 3 variables Pos assoc: 2 Neg assoc:1§ **100%**		3 articles 8 variables Pos assoc: 5 **63%**	2 articles 2 variables Pos assoc: 0 **0%**	17 articles 30 variables Pos assoc: 18 **60%**
**Outcome Expectations**	5 articles 13 variables Pos assoc: 6 **46%**			2 articles 2 variables Pos assoc: 1 **50%**					7 articles 15 variables Pos assoc: 7 **47%**
**Preferences**	13 articles 26 variables Pos assoc: 20 **77%**	2 articles 3 variables Pos assoc: 1 **33%**	5 articles 5 variables Pos assoc: 1 **20%**		1 article 1 variable Pos assoc: 0 **0%**		1 article 2 variables Pos assoc: 2 † **100%**		22 articles 37 variables Pos assoc: 24 **65%**
**Self-Efficacy**	14 articles 34 variables Pos assoc: 15 **44%**	4 articles 6 variables Pos assoc: 3 **50%**	5 articles 7 variables Pos assoc: 3 **43%**	2 articles 3 variables Pos assoc: 2 † **67%**	2 articles 2 variables Pos assoc: 1 **50%**		5 articles 9 variables Pos assoc: 6 **67%**	1 article 3 variables Pos assoc: 2 † **67%**	33 articles 64 variables Pos assoc: 32 **50%**
**Social Support**	6 articles 10 variables Pos assoc: 4 **40%**	4 articles 6 variables Pos assoc: 2 **33%**	3 articles 3 variables Pos assoc: 2 **67%**		1 article 1 variables Pos assoc: 0 **0%**	2 articles 4 variables Pos assoc: 4 **100%**	3 articles 8 variables Pos assoc: 5 **63%**	1 article 1 variable Neg assoc: 1 † **100%**	20 articles 33 variables Pos assoc: 18 **55%**

**‡ **Perceived availability was significant in 10 of the 12 articles, classifying it as consistently significant.

† Does not classify as consistent because association is only observed in one article.

§ This is a measure of milk norms and is therefore considered to represent the proper direction.

## Discussion

This review of the literature on psychosocial correlates of dietary intake in children and adolescents illustrates that perceived modeling and dietary intentions to make healthy or less healthy dietary changes (such as intentions to decrease consumption of sugary beverages or intentions to increase consumption of medium fat milk) have the most consistent and positive associations with eating behavior. Other psychosocial correlates such as liking, norms, and preferences were also consistently and positively associated with eating behavior in children and adolescents. However, somewhat surprisingly, availability, knowledge, outcome expectations, self-efficacy and social support did not show consistent relationships across dietary outcomes.

The recurring relationship between the psychosocial correlates of perceived modeling [19,23,24,114], norms [115], and preferences [19,22,114] in predicting eating behavior in children and adolescents is supported in other reviews [19,22-24,114,115]. For example, in a review that focused on the environmental correlates of obesity-related dietary behavior in youth, van der Horst et al. found the most consistent associations between parental intake and children's fat and F&V intake [23]. That review also found the most consistent association between parent and sibling intake with adolescents' energy and fat intake [23]. A review on determinants of fruit and vegetable intake by Rasmussen et al. also illustrated a positive association between parental intake and fruit and vegetable intake [19]. The current review supports modeling as one of the most powerful psychosocial predictors of dietary intake in youth. However, our review is unique because it illustrates that perceived modeling is the single most consistent correlate of eating behavior. Modeling as reported by a parent is not consistently correlated with dietary intake. Second, this review is unique in its finding that intentions was also a consistent correlate of eating behavior across various samples of children and adolescents and various dietary outcomes.

Our results are quite dissimilar to a recent review of predictors of dietary behavior (fruit and vegetable intake in particular) among adults [20], and this emphasizes the need to examine psychosocial correlates of youth separately from those of adults. The recent review among adults showed that knowledge, self-efficacy, and social support were consistent predictors of fruit and vegetable intake, while we found that these variables did not predict eating behavior in children and adolescents. This finding highlights the fact that findings from adult research do not necessarily apply to pediatric populations; thus it is imperative that research and interventions in youth be tailored specifically to that population.

Self-report food frequency questionnaires (FFQs) and other types of self-report questionnaires or screeners were the most frequently used methods to measure dietary intake in youth. Only 10% of studies used dietary recalls and 16% used dietary records, which are considered to be more valid means of assessing dietary intake [116,117]. FFQs delivered a higher percentage of significant correlations with psychosocial variables while stronger measures of dietary intake (i.e. dietary recalls or dietary records) resulted in lower frequency of correlation with psychosocial variables. Different methods of measuring dietary intake delivered different results. More research is needed in the implementation of different outcome measures.

Intentions, a cognitive variable, was the second most consistent variable in predicting eating behavior, which runs contrary to literature suggesting that children's behavior is driven by more affective, rather than cognitive determinants. However, the finding that knowledge and self-efficacy were not predictive of eating behavior in children suggests that children may not be as cognitive as their adult counterparts. This review was limited by the fact that there are few studies that incorporate theories of health behavior that focus on affective behavioral pathways. Therefore, assessment of affective predictors of health behaviors in pediatric populations remains limited. Further, most theory-based studies are based on cognitive theories of behavior. However, neurobiological evidence demonstrates age-related changes in cerebral functioning from lower-order, emotionally-based, sensory processing towards higher-order, more cognitive and rational, processing of stimuli by means of the prefrontal cortical systems that involve reward anticipation, self-monitoring, and behavioral inhibition [32]. Thus, the ability to make beneficial nutritional choices and regulate behavior may be affected by the neurobiological development of cognitive abilities that permit the inhibition of responses, delay of gratification and voluntary change of behavior to bypass short-term rewards in favor of longer-term goals [32]. This research supports the hypothesis that the cognitively-based health behavior models developed for adults may not be appropriate for adolescents who tend to be less rational, and more driven by affect. These findings illustrate that it is important to study children separately from adults and to focus on more affect driven psychological models.

### Limitations

Despite the strict protocol for assessing each article, there were some limitations to this study. Most of the studies (83%) included in this review were cross-sectional. Consequently, although such designs are useful in identifying possible theory-based associations, drawing conclusions about directionality and possible causality of associations is not possible. Cross-sectional designs can result in systematic error and an overestimation of associations between the psychosocial variables and eating behaviors [118].

The studies included in this review were diverse in the measurement of the psychosocial variables as well as dietary intake, samples, and analyses used. Therefore, it was not possible to conduct a true meta-analysis. Additionally, in order to summarize the findings of the studies, the authors combined conceptually similar psychosocial determinants into one category, which may have introduced bias.

There are also limitations to many of the studies reviewed here. If only the studies ranked as strong or exceptional were included in the review, twenty-six of the 77 articles would be excluded, resulting in a final inclusion of 51 articles. If only these 51 studies are included, norms are no longer associated with eating behavior. Additionally, there are no longer any articles to judge the association between outcome expectations and eating behavior. Besides these changes, the overall results of the review would remain the same. However, the review would be less reflective of the entire literature.

Additionally, most studies relied on self-report of dietary intake, which has been shown to be considerably unreliable, having high rates of mis-reporting, usually in the direction of under-reporting among youth [119]. Furthermore, bias might have been introduced due to possible lack of validity or reliability of both dietary and psychosocial measures. Unfortunately, reliability and validity of dietary and psychosocial measures were not reported in the some of the studies and only a few studies used objective observation to measure the dietary outcome. This illustrates the need for ongoing validity and reliability evaluation to ensure valid and reliable psychosocial and dietary outcome measures for use in cross-sectional as well as longitudinal studies.

Furthermore, certain studies reported only significant findings and did not address non-significant findings; thus there is a potential bias towards significant findings. Our search strategy also only included studies that were published in English in peer-reviewed journals and referenced in electronic databases; therefore, this review may have overlooked important studies published in other languages.

Lastly, this review did not separate children and adolescents into distinct categories, although research has suggested that children and adolescents exhibit different health behaviors [11]. However, separation by age-group yielded too few studies per variable per group to enable interpretation of findings.

### Implications and Future Directions

The goal of this literature review was to assess and report the most promising psychosocial correlates of eating behavior in children and adolescents in order to inform development of interventions. A major strength of this review is that it included a diverse and large sample of studies. For example, studies included samples from many different countries, thus enhancing the generalizability of findings to children and adolescents around the world. Because previous reviews have looked at a single eating behavior (e.g. FVJ consumption only) among adult populations [20], this study addresses a key gap in the literature to serve as a tool for behavioral theorists and interventionists by investigating the psychosocial correlates of diverse eating behaviors in children and adolescents. Strong evidence was found for intentions, perceived modeling, norms, liking, and preferences as consistent correlates of the eating behavior of children and adolescents across dietary outcomes. Comparison of the outcomes of this review paper with recent reviews of the adult literature suggests that determinants of eating behavior differ between adult and pediatric populations. For instance, among adults, knowledge and outcome expectations are consistent correlates of eating behaviors, while in children, they are not. This supports our earlier work showing that knowledge does not impact behavior consistently in youth [11]. A key finding from this review is that health behavior theory as well as research on affective pathways relevant to youth populations is lacking.

Future intervention research may benefit from the incorporation of findings from this review to create more effective adolescent and childhood dietary interventions by targeting the variables shown in this review that are most consistently associated with the various eating behaviors such as intentions, modeling, norms, liking, and preferences. Based on the fact that these constructs have repeatedly been found to be related to dietary behavior, intervening on these factors may be the best way to elicit dietary change. Future research should also investigate the variables that have been insufficiently examined to date, particularly the variables rooted in affective theories. It is quite plausible that affective factors, such as motivation [120], executive control [121], or meanings of behavior [122] might drive the dietary behavior of children and adolescents.

Lastly, future research should investigate the psychosocial correlates of several dietary behaviors that are known to influence weight and metabolic health such as fat and fiber [123] that have been understudied.

## Conclusion

This review examined the correlates of various dietary intake; Fruit, Juice and Vegetable Consumption, Fat in Diet, Total Energy Intake, Sugar Snacking, Sweetened Beverage Consumption, Dietary Fiber, Other Healthy Dietary Consumption, and Other Less Healthy Dietary Consumption in cross-sectional and prospective studies for children and adolescents. The correlates most consistently supported by evidence were perceived modeling, dietary intentions, norms, liking and preferences. More prospective studies on the psychosocial determinants of eating behavior and broader theoretical perspectives should be examined in future research.

## Competing interests

The authors declare that they have no competing interests.

## Authors' contributions

ADM carried out the literature review, data extraction, conceptualization, interpretation of the data, and drafted the manuscript. CC participated in the literature review, data extraction, and interpretation of the data. STN participated in the data extraction, interpretation of the data and helped to draft the manuscript. ALY contributed to the conceptualization, data interpretation, and drafting of the manuscript. DSM participated in the conceptualization, data extraction, interpretation of the data and drafting of the manuscript. All authors read and approved the final manuscript.
